# Galcanezumab in episodic migraine: subgroup analyses of efficacy by high versus low frequency of migraine headaches in phase 3 studies (EVOLVE-1 & EVOLVE-2)

**DOI:** 10.1186/s10194-019-1024-x

**Published:** 2019-06-28

**Authors:** Stephen D. Silberstein, Virginia L. Stauffer, Katie A. Day, Sarah Lipsius, Maria-Carmen Wilson

**Affiliations:** 1 Jefferson Headache Center, Thomas Jefferson University, Philadelphia, PA USA; 2 Lilly Research Laboratories, Lilly Corporate Center, Indianapolis, Indiana USA; 3 Syneos Health, Raleigh, NC USA; 4 Ochsner Health System, Covington, LA USA

**Keywords:** Episodic migraine, Migraine frequency, Galcanezumab

## Abstract

**Background:**

Patients with high-frequency episodic migraine (HFEM) have a greater disease burden than those with low-frequency episodic migraine (LFEM). Acute treatment overuse increases the risk of migraine chronification in patients with HFEM. Galcanezumab, a humanized monoclonal antibody binding calcitonin gene-related peptide (CGRP), is effective for migraine prevention with a favorable safety profile. Here, we investigate whether there are differences in galcanezumab efficacy in patients with LFEM or with HFEM.

**Methods:**

Data were pooled from two double-blind, placebo-controlled phase 3 trials; EVOLVE-1 and EVOLVE-2. Patients were 18–65 years old, experienced 4–14 monthly migraine headache days (MHDs) for ≥1 year prior, with onset at < 50 years of age. Migraine headaches were tracked via electronic patient-reported outcome system and randomization was stratified by low (LFEM; 4–7 monthly MHDs) or high (HFEM; 8–14 monthly MHDs) frequency. Subgroup analysis compared the HFEM and LFEM subgroups with a linear or generalized linear mixed model repeated measures approach.

**Results:**

The intent-to-treat patients (*N* = 1773) had a mean age of 41.3 years, were mostly white (75%), female (85%), and 66% of patients had HFEM. In both the LFEM and HFEM subgroups, the overall (Months 1–6) and monthly changes from baseline in monthly MHDs and monthly MHDs with acute medication use compared with placebo were statistically significantly reduced for galcanezumab 120-mg and 240-mg. Galcanezumab (120-mg and 240-mg) significantly decreased the overall and monthly MHDs with nausea and/or vomiting, and with photophobia and phonophobia versus placebo in patients with LFEM or HFEM. In both subgroups, the mean overall (Months 1–6) and monthly percentages of patients with ≥50%, ≥75%, and 100% reduction in monthly MHDs from baseline were statistically significantly greater in patients receiving either dose of galcanezumab versus placebo. Galcanezumab (120-mg and 240-mg) significantly improved the Migraine-Specific Quality of Life Questionnaire role function-restrictive domain score as well as the Migraine Disability Assessment total score versus placebo for patients with LFEM or HFEM. There were no significant subgroup-by-treatment interactions.

**Conclusions:**

Galcanezumab was as effective in patients with HFEM as in those with LFEM. Associated symptoms, quality of life, and disability were similarly improved in patients with HFEM or LFEM.

**Trial registration:**

NCT02614183, NCT02614196.

## Background

Migraine is the second global cause of years lived with disability (YLDs), representing 45.1 million (95% uncertainty interval [95%UI]: 29.0–62.8 million) YLDs [1]. Its global age-standardized prevalence of 18.9% (95%UI: 18.1–19.7%) for women and 9.8% (95%UI: 9.4–10.2%) for men represents a world-wide total of 1.04 billion (95%UI: 1.00–1.09 billion) individuals with migraine [2]. Despite this, it is often under-treated and inadequately recognized [1–3].

Migraine frequency varies. While episodic migraine (EM) is not specifically defined in the 3rd edition of the *International Classification of Headache Disorders* (ICHD) [4], it refers to a headache frequency of less than 15 headache days per month in patients who have migraine [3]. The ICHD-3 defines chronic migraine (CM) as 15 or more headache days per month with at least 8 days meeting ICHD criteria for migraine with or without aura [4]. Although using 15 days per month as a divide between EM and CM appears arbitrary, there are meaningful differences in epidemiology, as CM may be more difficult to treat, associated with more comorbidities, with more severe and longer-lasting migraine headaches, and greater functional impact than EM [3]. Approximately 2% to 3% of patients with EM progress to CM annually [3, 5], and in one study, 26% of patients with CM reverted to EM over 2 years [5]. This suggests there may be some overlap in biology between CM and EM experienced at higher frequencies; consequently, there may be differences in the responses of patients with high-frequency EM (HFEM) compared to those with low-frequency EM (LFEM).

Unlike CM, there is no standardized definition of LFEM and HFEM, and different studies have used frequencies from 8 to 14 and 10 to 14 migraine headache days (MHDs) per month to define HFEM [6–8]. The recent approvals of monoclonal antibodies to calcitonin gene-related peptide (CGRP), fremanezumab and galcanezumab, along with approval of monoclonal antibodies to the CGRP receptor, erenumab, for migraine prevention open a new chapter in the management of EM and CM. The present investigation was undertaken to compare the effect of galcanezumab to placebo in patients with LFEM and HFEM to assess if the treatment effect of galcanezumab differed in these 2 subgroups of patients categorized by migraine headache frequency.

## Methods

### Study design

Patient data were pooled from 2 phase 3 multicenter, placebo-controlled, randomized clinical trials, EVOLVE-1 (NCT02614183) and EVOLVE-2 (NCT02614196), designed to examine the ability of galcanezumab to reduce the monthly number of MHDs in patients with episodic migraine. EVOLVE-1 was conducted at 90 study sites in the United States and Canada, and EVOLVE-2 was conducted at 109 study sites in the United States, United Kingdom, Netherlands, Spain, Czech Republic, Germany, Argentina, Israel, Korea, Taiwan, and Mexico. The details of the clinical trials have been published [9, 10]. The study protocols were reviewed and approved by the appropriate institutional review board for each of the study sites. All patients gave written informed consent to participate in the study. The studies were conducted with the approval of the independent Ethics Committees of the participating institutes and in accordance with the Declaration of Helsinki, the International Conference on Harmonization Good Clinical Practice guidelines, and local regulations.

Both trials consisted of 2 study periods prior to the randomization step. Study period 1 (3 to 45 days) consisted of a comprehensive medical examination and wash-out of migraine preventive medications. Study period 2 (30–40 days) was the prospective baseline period, when patients logged in daily to the electronic patient-reported outcomes (ePRO) system and reported on the occurrence of headaches, headache duration, headache features, severity of headache, and use of headache medication in order to confirm migraine headache frequency. Study period 3 (6 months) was the double-blind treatment phase, where patients were randomized (2:1:1) to receive subcutaneous injections of either placebo, 120-mg galcanezumab, or 240-mg of galcanezumab during monthly office visits. Patients in the 120-mg galcanezumab group received an initial loading dose of 240-mg. Patients in all groups received 2 injections at each dosing visit in order to preserve blinding throughout the study. Patients continued daily-diary entries and were permitted to take acute migraine medications as needed. Medications containing opioids or barbiturates were limited to three days monthly and other migraine preventive treatments were excluded. Randomization was stratified by country and migraine frequency (< 8 vs ≥8 MHDs/month) at baseline in order to achieve balance among groups.

Patients enrolled in the study were between the ages of 18 and 65 years and had a diagnosis of migraine with or without aura [11] for ≥1 year prior to enrollment and onset prior to the age of 50 years. In order to be included in the study, patients also had to have 4 to 14 MHDs per month and at least 2 migraine episodes during the prospective baseline period, as well as 80% compliance in using the electronic diary.

Patients were excluded if they participated in a clinical trial within the previous 30 days, were previously exposed to a CGRP antibody, including galcanezumab, or had known hypersensitivity to multiple drugs, monoclonal antibodies or other therapeutic proteins. Patients with a history of persistent daily headache, cluster headache, or migraine subtypes (hemiplegic, ophthalmoplegic, or migraine with brainstem aura, or chronic migraine) as defined by ICHD-3 β [11], as well as those previously failing to respond to ≥3 migraine preventive treatments from different therapeutic classes, or presence of a medical condition that would preclude study participation including but not limited to pregnancy, suicidal ideation within the past month, history of substance abuse or dependence in the past year, recent history of acute cardiovascular events, and/or serious cardiovascular risk based on history or electrocardiogram findings were excluded. Patients who used opioids or barbiturate-containing analgesics more than twice per month in more than 2 of the past 6 months were also excluded, as were those who were pregnant.

### Assessments used

Efficacy analyses were performed on the intent-to-treat (ITT) population, which included randomized patients who received at least one dose of galcanezumab or placebo. Assessments were based on the responses made by the patients in the ePRO daily diary. An automated algorithm was used to define each day as either a migraine day, probable migraine day (headache with or without aura lasting ≥30 min), non-migraine headache day, or no headache day. A MHD was defined as a calendar day on which a migraine headache or probable migraine headache occurred. The number of monthly MHDs and number of monthly MHDs with acute medication use, with aura, with nausea and/or vomiting, with photophobia and phonophobia, and with prodrome for Months 1 to 6 were determined from the ePRO data. In addition, the Migraine-Specific Quality of Life Questionnaire v2.1 Role Function-Restrictive (MSQ-RFR v2.1) assessments were performed during the monthly visits at each study site. The Migraine Disability Assessment (MIDAS) was performed at Months 3 and 6.

### Statistical analysis

The frequency of episodic migraine headaches was determined for each patient during the prospective baseline. Patients were then categorized post-hoc into LFEM (4–7 MHD) or HFEM (8–14 MHD) monthly migraine frequency subgroups. The baseline symptoms, functioning, and disability for the 2 migraine frequency subgroups were compared using an Analysis of Variance model with terms for study and baseline migraine frequency group in order to determine if illness burden was impacted by monthly migraine frequency. Analyses within subgroups were conducted for continuous efficacy measures using a mixed models repeated measures approach with terms for treatment, pooled country (study), month, and treatment-by-month interaction, baseline value, and baseline-by-month interaction. The model used to compare subgroups had additional terms for subgroup, subgroup-by-treatment, subgroup-by-month, and subgroup-by treatment-by-month interaction. The primary endpoint of overall mean change in monthly MHDs was the average of values obtained at Months 1 to 6 of the 6-month double-blind treatment phase.

Change from baseline of continuous variables (i.e., MHD, MHD with acute medication use, MHD with nausea and/or vomiting, MHD with photophobia and phonophobia, MSQ-RFR and Patient Global Impression of Severity [PGI-S]) were analyzed by month and overall (for Months 1 to 6); MSQ-RFR and PGI-S were also analyzed overall (for Months 4 to 6). The efficacy measures of ≥50%, ≥75%, and 100% reduction from baseline (“response”) in the number of monthly MHDs were analyzed within each subgroup by month and overall (Months 1 to 6) using a categorical, pseudo-likelihood-based repeated measures model for binary outcomes with terms for treatment, study, month, treatment*month, and baseline MHD. Subgroups were compared using this model with additional terms for subgroup, subgroup-by-treatment, subgroup-by-month, and subgroup-by-treatment-by-month interaction.. Treatment effects were evaluated based upon a two-sided, 0.05 significance level. Effects for continuous (binary) outcomes are presented using model estimated means (proportions) while differences in continuous outcomes between groups were shown using mean differences along with 95% confidence intervals. The subgroup-by-treatment interaction was tested at a two-sided, 0.1 significance level. All statistical analyses were performed with the use of SAS software version 9.4 (SAS Institute, Cary, NC).

## Results

### Patient disposition and baseline characteristics

A total of 1773 patients were included in the ITT population, with 597 (34%) in the LFEM group and 1176 (66%) in the HFEM group. Overall, the patient population was largely female (84.6%), white (75.2%), and North American (73.6%), and had a mean time since migraine diagnosis of 20.3 years. The mean (standard deviation [SD]) age of the patients was 41.3 (11.4) years, and there was no significant difference in age between the LFEM and HFEM groups. At baseline, patients with HFEM had significantly more MHDs, and MHDs with acute medication use, with nausea and/or vomiting, with photophobia and phonophobia, with aura, and with prodromal symptoms than did patients with LFEM. Patient baseline characteristics are summarized in Table 1.

**Table 1:** Baseline characteristics

	LFEM Mean (SD)	HFEM Mean (SD)	LFEM vs HFEM *p*-value *
Number of patients (%)	597 (33.7)	1176 (66.3)	
Age at randomization, years	42 (11.4)	41 (11.4)	.069
MHDs	5.79 (1.11)	10.82 (2.01)	<.001
MHDs with acute medication use	4.82 (1.73)	8.81 (3.27)	<.001
MHDs with nausea and vomiting	2.20 (1.98)	4.66 (3.36)	<.001
MHDs with photophobia and phonophobia	4.08 (2.17)	8.26 (3.52)	<.001
MHDs with aura	1.40 (2.03)	3.04 (3.65)	<.001
MHDs with prodrome	1.89 (2.11)	3.86 (3.77)	<.001
MSQ-RFR score	55.8 (15.8)	49.5 (15.4)	<.001
MIDAS total score	26.3 (22.9)	36.6 (30.7)	<.001

* P-value from Analysis of Variance model with terms for study, baseline MHD frequency subgroup

HFEM: high-frequency episodic migraine; ITT: intent-to-treat; LFEM: low-frequency episodic migraine; MHDs: migraine headache days; MIDAS: Migraine Disability Assessment; MSQ-RFR v2.1: Migraine-Specific Quality of Life Questionnaire v2.1 Role Function-Restrictive

### Change in monthly MHDs

**Fig. 1:**
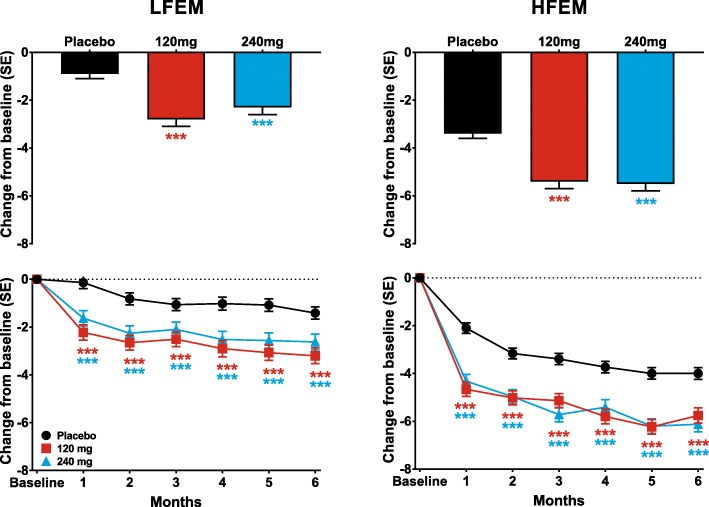
The overall least-squares (LS) mean change from baseline in monthly MHDs is shown for patients with LFEM and HFEM receiving placebo, 120-mg, or 240-mg of galcanezumab in the upper row. The monthly LS mean changes in monthly MHDs for patients receiving these treatments is shown in the bottom row for patients with LFEM and with HFEM. ***p ≤ .001, **p ≤ .01, *p ≤ .05 vs placebo

Galcanezumab 120-mg and 240-mg significantly reduced the mean number of monthly MHDs over the 6-month treatment period in patients with LFEM and in those with HFEM compared to placebo (Table 2). These reductions represent changes of − 1.8 (95% confidence intervals [CI]: − 2.35, − 1.32) and − 1.4 (95% CI: − 1.88, − 0.84) in monthly MHDs relative to placebo for 120-mg and 240-mg of galcanezumab, respectively, for patients with LFEM and − 2.0 (95% CI: − 2.56, − 1.52) and − 2.1 (95% CI: − 2.58, − 1.54) in monthly MHDs relative to placebo for 120-mg and 240-mg of galcanezumab, respectively, for patients with HFEM. It is noteworthy that the differences relative to placebo are similar, regardless of migraine frequency, and reflect the higher placebo response seen in patients with HFEM. Both doses of galcanezumab significantly reduced the numbers of MHDs at each month over the 6-month treatment period relative to reductions in the placebo groups of patients with LFEM and HFEM (Fig. 1). The treatment-by-subgroup interaction was not statistically significant indicating that the treatment effect did not differ across the LFEM and HFEM subgroups.

**Table 2:** Reduction over Months 1 to 6 in mean monthly MHDs with associated symptoms

	LFEM Average of Months 1–6 (95% CI)			HFEMAverage of Months 1–6 (95% CI)		
Monthly MHDs with:	Placebo (*N* = 295)	GMB 120 mg (*N* = 150)	GMB 240 mg (*N* = 145)	Placebo (*N* = 580)	GMB 120 mg (*N* = 286)	GMB 240 mg (*N* = 283)
All MHDs	−0.9 (−1.4,-0.5)	−2.8 (−3.3, − 2.2)	−2.3 (− 2.8, − 1.7)	−3.4 (− 3.8, 3.0)	−5.4 (− 5.9, − 4.9)	−5.5 (−6.0, − 4.9)
p vs placebo		<.001	<.001		<.001	<.001
Acute medication use	− 0.8 (− 1.2, − 0.4)	− 2.4 (− 2.9, − 2.0)	−2.1 (− 2.6, − 1.7)	−2.7 (− 3.1, − 2.7)	−4.6 (− 5.1, − 4.2)	−4.6 (− 5.0, − 4.2)
p vs placebo		<.001	<.001		<.001	<.001
Prodromal symptoms	−0.6 (− 0.9, − 0.4)	−1.1 (− 1.4, − 0.7)	−1.1 (− 1.4, − 0.7)	−1.4 (− 1.6, − 1.1)	− 2.2 (− 2.6, − 1.9)	−2.0 (− 2.4, − 1.7)
p vs placebo		.006	.006		<.001	<.001
Aura	−0.4 (− 0.6, − 0.2)	−0.8 (− 1.0, − 0.5)	−0.7 (− 0.9, − 0.4)	− 1.3 (− 1.5, − 1.0)	−1.8 (− 2.0, − 1.5)	−1.8 (− 2.1, − 1.5)
p vs placebo		.005	.055		<.001	<.001
Photophobia and phonophobia	−0.5 (− 0.9, − 0.1)	−1.9 (− 2.4, − 1.4)	−1.7 (− 2.2, − 1.2)	− 2.5 (− 2.9, − 2.1)	− 4.2 (− 4.7, − 3.7)	−4.1 (− 4.6, − 3.6)
p vs placebo		<.001	<.001		<.001	<.001
Nausea and/or vomiting	−0.2 (− 0.5, 0.1)	−0.9 (− 1.2, − 0.6)	−0.9 (− 1.2, − 0.6)	−1.5 (− 1.8, − 1.2)	−2.6 (− 2.9, − 2.2)	−2.4 (− 2.8, − 2.1)
p vs placebo		<.001	<.001		<.001	<.001

Abbreviations: 95% CI: 95% Confidence interval; GMB: galcanezumab; HFEM: high-frequency episodic migraine; LFEM: low-frequency episodic migraine; MHD: migraine headache day

### Change in monthly MHDs with acute medication use

Galcanezumab 120-mg and 240-mg significantly reduced the mean number of monthly MHDs with acute medication use over the 6-month treatment period in patients with LFEM or HFEM compared to placebo (Table 2). For the patients with LFEM, these changes represent reductions of 1.7 (95% CI: − 2.11, − 1.20) and 1.4 (95% CI: − 1.82, − 0.89) in MHDs beyond those due to placebo, and reductions of 1.9 (95% CI: − 2.35, − 1.45) and 1.9 (95% CI: − 2.35, − 1.43) MHDs relative to placebo for both doses in patients with HFEM. Both doses of galcanezumab showed significant reductions relative to placebo in numbers of MHDs with acute medication use at each month over the 6-month treatment period in patients with LFEM and HFEM (Table 2). The treatment effect was consistent across the LFEM and HFEM subgroups as evidenced by the non-significant treatment-by-subgroup interaction.

### Change in monthly MHDs with symptoms associated with migraine

Galcanezumab 120-mg and 240-mg significantly reduced the mean numbers of monthly MHDs with aura, prodrome symptoms, nausea and/or vomiting, and with photophobia and phonophobia over the 6-month treatment period in patients with LFEM and with HFEM compared to placebo (Table 2). For the patients with LFEM, these changes ranged from − 0.7 (95% CI: − 0.9, − 0.4) MHDs, for days with aura, to − 1.9 (95% CI: − 2.4, − 1.4) days with photophobia and phonophobia. Likewise for patients with HFEM, the change in MHDs with associated symptoms ranged from − 1.8 (95% CI: − 2.0, − 1.5) MHDs with aura to − 4.2 (95% CI: − 4.7, − 3.7) MHDs with photophobia and phonophobia (Table 2). The treatment-by-subgroup interaction was not statistically significant indicating consistency of the treatment effect across the subgroups.

### Response rates in patients with LFEM and HFEM

**Fig. 2:**
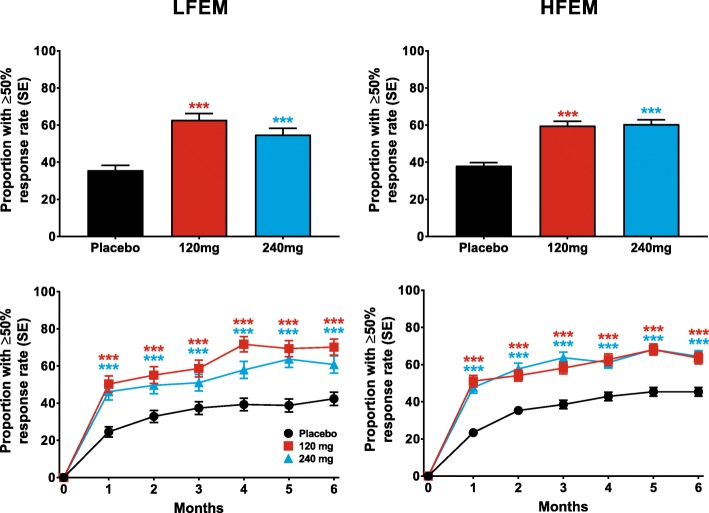
The overall mean percentage of patients with ≥50% reduction from baseline in monthly MHDs across Months 1–6 is shown for patients with LFEM and HFEM receiving placebo, 120-mg, or 240-mg of galcanezumab in the upper row. The monthly percentage of patients with ≥50% reduction in MHDs is shown in the bottom row for patients with LFEM and with HFEM. ***p ≤ .001, **p ≤ .01, *p ≤ .05 vs placebo

**Fig. 3:**
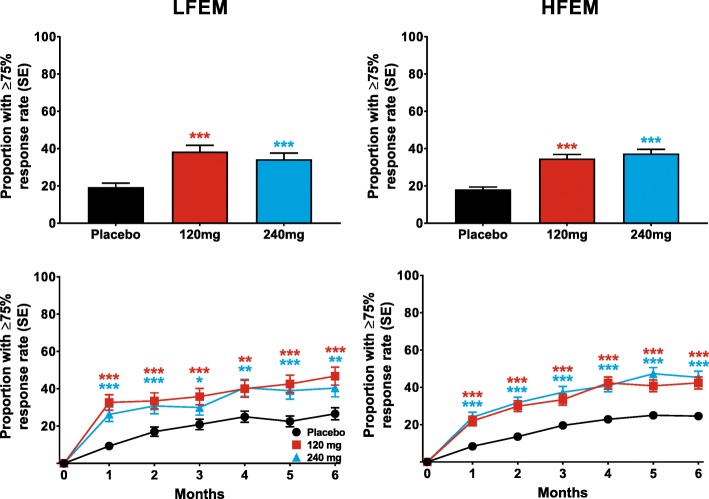
The overall mean percentage of patients with ≥75% reduction from baseline in monthly MHDs across Months1–6 is shown for patients with LFEM and HFEM receiving placebo, 120-mg, or 240-mg of galcanezumab in the upper row. The monthly percentage of patients with ≥75% reduction in MHDs is shown in the bottom row for patients with LFEM and with HFEM. ***p ≤ .001, **p ≤ .01, *p ≤ .05 vs placebo

**Fig. 4:**
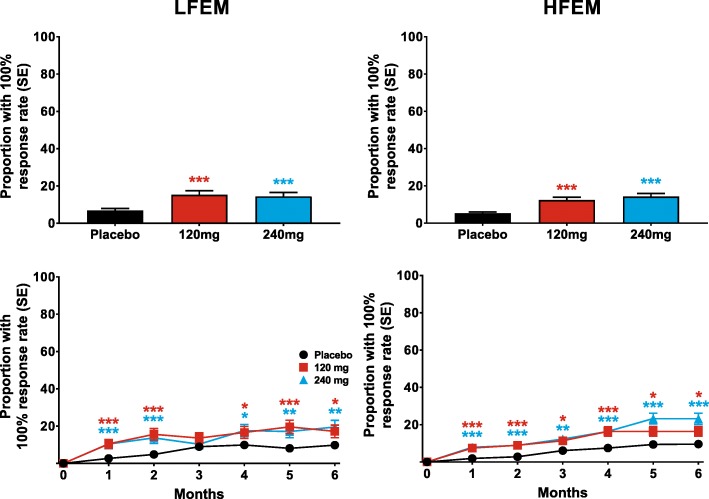
The overall mean percentage of patients with 100% reduction in monthly MHDs across Months 1–6 is shown for patients with LFEM and HFEM receiving placebo, 120-mg, or 240-mg of galcanezumab in the upper row. The monthly percentage of patients with 100% reduction in MHDs is shown in the bottom row for patients with LFEM and with HFEM. ***p ≤ .001, **p ≤ .01, *p ≤ .05 vs placebo

Both doses of galcanezumab produced similar ≥50% response rates overall (for Months 1–6), that were statistically significantly greater than placebo, in patients with LFEM or HFEM (Fig. 2). The overall estimated ≥50% response rate (standard error [SE]) over the 6-month treatment period was 63% (3%) and 55% (3%) for patients with LFEM who were treated with 120-mg and 240-mg of galcanezumab, respectively (Fig. 2). The average response rates over the same period for patients with HFEM treated with 120-mg and 240-mg of galcanezumab, respectively, were 60% (2%) and 61% (2%). The percentage of patients with ≥50% response rates was significantly greater than placebo at each month for both doses of galcanezumab in both the LFEM and HFEM groups (Fig. 2). Likewise, both doses of galcanezumab resulted in ≥75% (Fig. 3) and 100% (Fig. 4) response rates that were significantly greater than those of placebo in both the LFEM and HFEM groups. As with the 50% response rate, the proportion of patients with a ≥ 75% response did not differ between the LFEM and HFEM groups. Likewise, the mean over Months 1 to 6 of the percentage of patients with a 100% response rate was also similar between the 2 EM frequency subgroups. The percentage of patients with a ≥ 75% response rate (Fig. 3) and a 100% response rate (Fig. 4) at each month who received galcanezumab was significantly greater than the percentage achieving these response rates with placebo within each of the EM frequency subgroups. There was a statistically significant pairwise subgroup-by-treatment interaction (*p* = .09) for the ≥50% response rate between galcanezumab 120-mg and placebo for the overall (Months 1–6) time period. However, this interaction was deemed spurious and not meaningful as neither the subgroup-by-treatment interaction for galcanezumab 240-mg versus placebo nor the overall interaction were statistically significant.

**Fig. 5:**
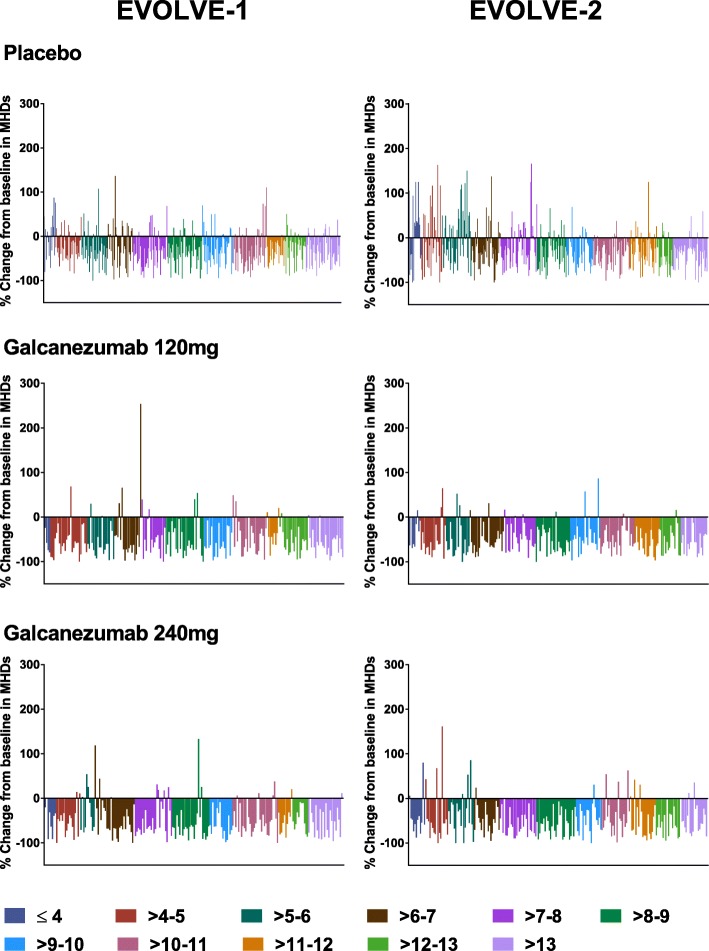
Waterfall plots showing the percent change from baseline in number of MHDs of Month 1 to 6 by baseline number of migraine headache days

Waterfall plots showing results for the primary endpoint by number of migraine days at baseline are presented in Fig. 5 by individual study (EVOLVE-1 and EVOLVE-2). These plots provide the percentage change from baseline in MHDs for each patient by baseline number of MHDs, and are color-coded for ranges of baseline MHDs. For galcanezumab-treated patients, there was no discernible difference in pattern between patients with the most and least MHDs at baseline.

### Disability and quality of life

**Fig. 6:**
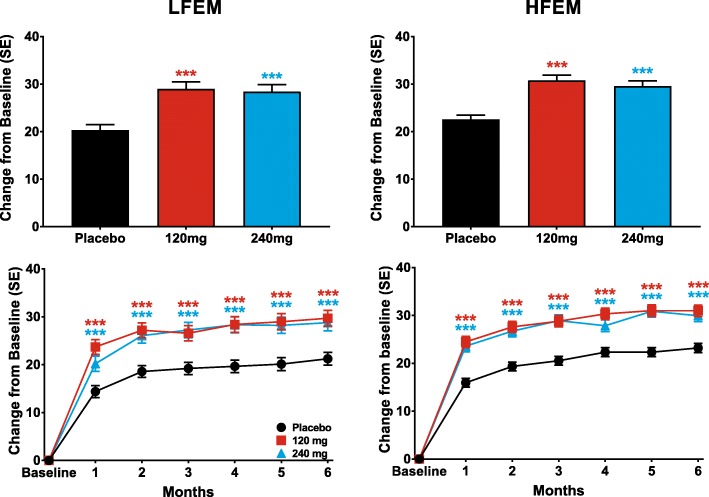
The overall least-squares (LS) mean change in the Migraine-Specific Quality of Life Questionnaire role function-restrictive domain (MSQ-RFR) is shown for patients with LFEM and HFEM receiving placebo, 120-mg, or 240-mg of galcanezumab in the upper row. The monthly LS mean changes in MSQ-RFR for patients receiving these treatments is shown in the bottom row for patients with LFEM and with HFEM. ***p ≤ .001, **p ≤ .01, *p ≤ .05 vs placebo

Galcanezumab 120-mg and 240-mg produced similar increases in MSQ-RFR scores overall at Months 4 to 6 in patients with both LFEM and with HFEM (Fig. 6). For both the LFEM and HFEM groups, with both doses of galcanezumab, the increases in MSQ-RFR scores were significantly greater than those of the placebo group at all visits throughout the 6-month treatment period (Fig. 6).

**Fig. 7:**
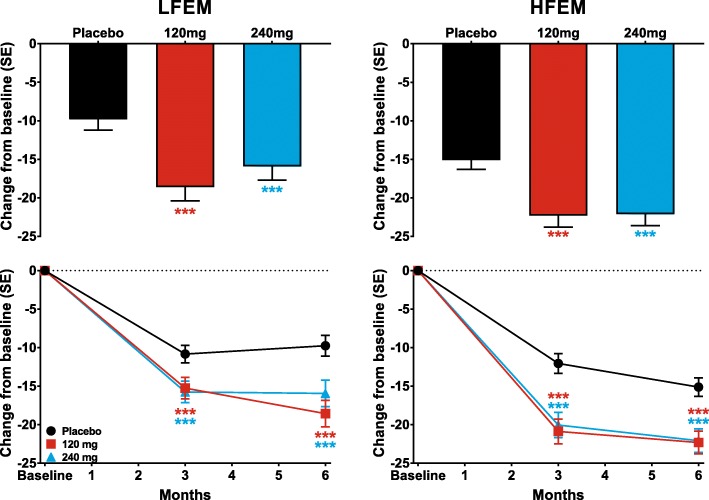
The overall least-squares (LS) mean change in Migraine Disability Assessment (MIDAS) total Score for the average of Months 3 and 6 is shown for patients with LFEM and HFEM receiving placebo, 120-mg, or 240-mg of galcanezumab in the upper row. The LS mean changes in MIDAS total score at Months 3 and 6 for patients receiving these treatments is shown in the bottom row for patients with LFEM and with HFEM. ***p ≤ .001, **p ≤ .01, *p ≤ .05 vs placebo

Likewise, mean reductions in the MIDAS total scores overall (average of Months 3 and 6), and at Months 3 and 6, were significantly greater than those in the placebo group for both doses of galcanezumab for patients with LFEM or with HFEM (Fig. 7). Treatment-by-subgroup interactions were not statistically significant for MSQ-RFR and MIDAS total score confirming consistency of treatment effect across the LFEM and HFEM subgroups.

## Discussion

Galcanezumab given in monthly doses of 120-mg and 240-mg was effective in reducing the numbers of MHDs in patients with LFEM and with HFEM. Moreover, as the waterfall plots showed, galcanezumab was effective regardless of the baseline frequency of MHDs. Change relative to the patient’s baseline is a useful measure of comparison across baseline groupings since a reduction of, for example, 5 MHDs could represent 100% response for a patient with 5 MHDs at baseline but less than 50% response for one with 14 MHDs at baseline. The plots of percentage change in the galcanezumab treatment groups show that for every grouping by baseline number of MHDs, there was a similar set of results, with a few patients in most groups showing worsening from baseline, but a substantial number of patients in each group showing clinically significant improvement from baseline (> 50% reduction in MHDs relative to baseline) for the episodic migraine studies. In addition, galcanezumab reduced the monthly MHDs with acute medication use, with prodromal symptoms other than aura, with aura, with nausea and/or vomiting, and with photophobia and phonophobia, by similar degrees in patients with LFEM and with HFEM. Migraine had a significantly greater impact on the quality of life and disability of patients with HFEM than those with LFEM, as indicated by the significant differences in MSQ-RFR and MIDAS scores at baseline. Both doses of galcanezumab produced similar levels of improvement in both the MSQ-RFR and MIDAS total scores in patients with LFEM and with HFEM. These results suggest that galcanezumab is equally effective in patients with episodic migraine, regardless of headache frequency.

In addition to migraine headache frequency, migraine severity, migraine duration, level of disability and symptoms, the impact on patient, and response to acute treatment are important factors for the initiation of preventive treatment [12]. The advisory group for the American Migraine Prevalence and Prevention study recommended that preventive therapy be offered to migraine patients reporting either ≥6 MHDs per month, ≥4 MHDs per month with some impairment, or ≥ 3 MHDs per month with severe impairment or requiring bed rest [13]. Until the recent development of the monoclonal antibodies to CGRP or the CGRP receptor, migraine preventive therapies have not been migraine-specific, and have often had adverse events and poor patient compliance [3, 14, 15].

The recently approved monoclonal antibodies to CGRP or its receptor, fremanezumab, galcanezumab, and erenumab, as well as eptinezumab which is still in development, have been reported as effective in episodic migraine, are well tolerated, and have few adverse events [9, 10, 16–20]. The most commonly reported adverse events associated with galcanezumab in the EVOLVE-1 and EVOLVE-2 studies were injection site pain and associated injection site reactions [9,10,]. Moreover, in a recent phase 3 randomized clinical trial, galcanezumab was effective in patients with chronic migraine, and the most common adverse events were related to injection site adverse events [21].

A potential limitation of the present study is that for both EVOLVE-1 and EVOLVE-2, the definition of a MHD included the presence of migraine headache and probable migraine headache [9, 10]. This definition may make direct comparisons to literature guidelines based solely on migraine headache days, exclusive of probable migraine, somewhat difficult.

## Conclusions

In summary, once-monthly galcanezumab reduces the frequency of migraine headache days in patients with LFEM or HFEM. The numbers of days with acute medication use are also reduced, which helps lower the risk of developing medication-overuse headache. Reduction in MHDs with associated symptoms of nausea and vomiting, or photophobia and phonophobia, along with the changes in MSQ-RFR and MIDAS scores, suggest that galcanezumab treatment reduces the disability caused by migraine and increases quality of life in patients with either LFEM or HFEM.

## Data Availability

The data that support the findings of this study are available from the corresponding author upon reasonable request.

## Abbreviations

95% UI: 95% uncertainty interval
CGRP: Calcitonin gene-related peptide
CM: Chronic migraine
EM: Episodic migraine
ePRO: electronic patient-reported outcomes
HFEM: High-frequency EM
ICHD: International Classification of Headache Disorders
ITT: Intent-to-treat
LFEM: low-frequency EM
MHD: migraine headache day
MIDAS: Migraine Disability Assessment
MSQ-RFR v2.1: Migraine-Specific Quality of Life Questionnaire v2.1 Role Function-Restrictive
PGI-S: Patient Global Impression of Severity
SD: Standard deviation
SE: Standard error
YLDs: Years lived with disability
