# Correction to: Galcanezumab in episodic migraine: subgroup analyses of efficacy by high versus low frequency of migraine headaches in phase 3 studies (EVOLVE-1 & EVOLVE-2)

**DOI:** 10.1186/s10194-019-1069-x

**Published:** 2019-12-27

**Authors:** Stephen D. Silberstein, Virginia L. Stauffer, Katie A. Day, Sarah Lipsius, Maria-Carmen Wilson

**Affiliations:** 1 Jefferson Headache Center, Thomas Jefferson University, Philadelphia, PA USA; 2 Lilly Research Laboratories, Lilly Corporate Center, Indianapolis, Indiana USA; 3 Syneos Health, Raleigh, NC USA; 4 Ochsner Health System, Covington, LA USA

**Correction to: J Headache Pain**

**https://doi.org/10.1186/s10194-019-1024-x**

After publication of our article [1] we were notified that the data presented in the upper row of Fig. 7 was inadvertently the least square mean change from baseline (standard error) at Month 6 rather than the overall average of Month 3 and Month 6. The figure legend and discussion of the data in the text were and are correct. The error was only in the upper row of Fig. 7. The legend for Fig. 7 did not require revision.

**Fig. 7:**
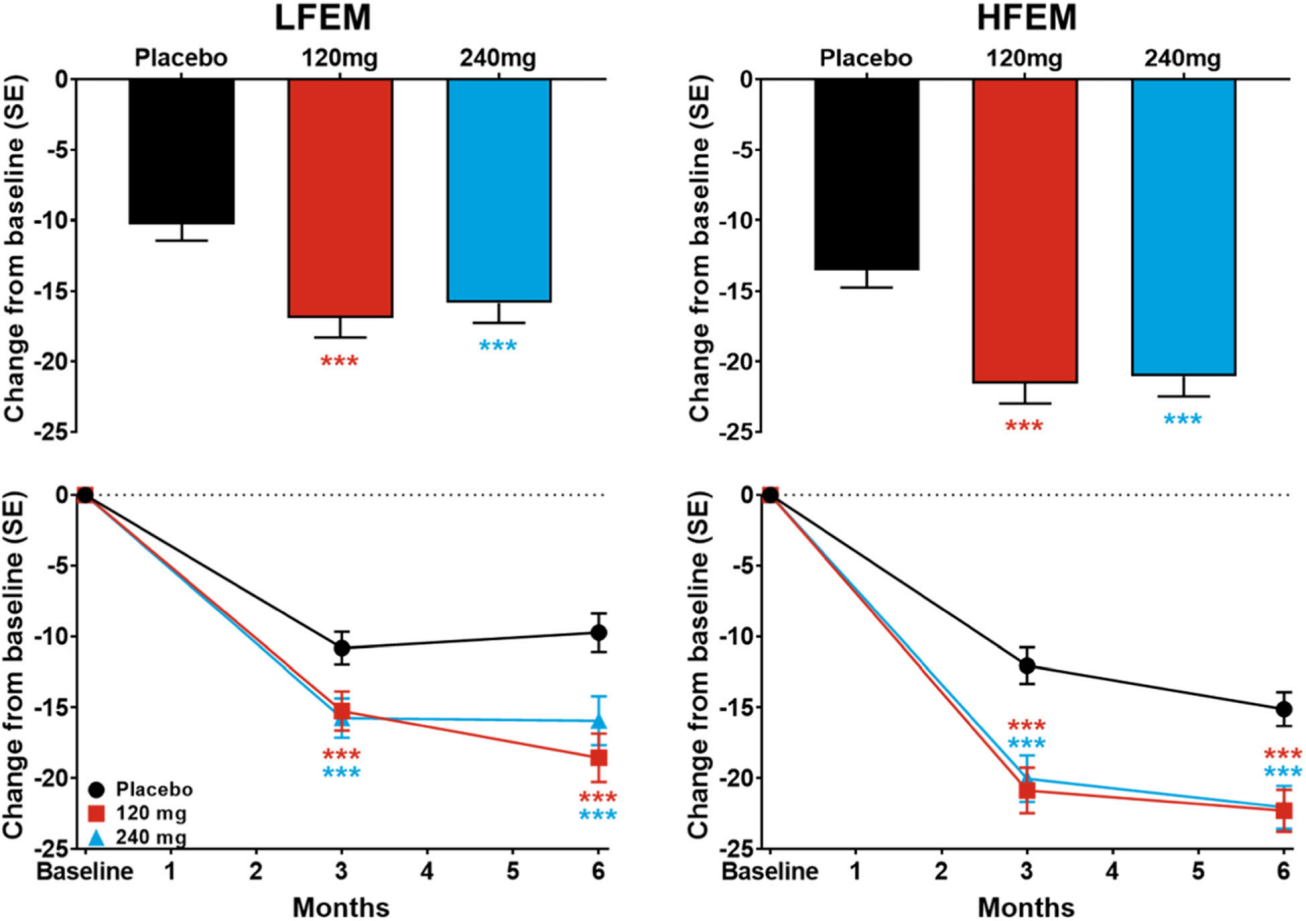
The overall least-squares (LS) mean change in Migraine Disability Assessment (MIDAS) total Score for the average of Months 3 and 6 is shown for patients with LFEM and HFEM receiving placebo, 120-mg, or 240-mg of galcanezumab in the upper row. The LS mean changes in MIDAS total score at Months 3 and 6 for patients receiving these treatments is shown in the bottom row for patients with LFEM and with HFEM. ***p ≤ .001, **p ≤ .01, *p ≤ .05 vs placebo
